# Attrition as one of the challenges of developing a palliative care centre: experience of the Indian Institute of Head and Neck Oncology, Indore, India

**DOI:** 10.3332/ecancer.2018.ed80

**Published:** 2018-03-12

**Authors:** Digpal Dharkar

**Affiliations:** Indore Cancer Foundation Charitable Trust, Indian Institute of Head and Neck Oncology, Pigdamber Road, District Indore 453331, India

**Keywords:** palliative care, attrition, India

## Abstract

Palliative care is an essential component of health care delivery. With respect to India, currently, despite rapid socio economic development, there are tremendous challenges in offering institutional palliative care due to several factors. A major factor has been an acute shortage of trained palliative care professionals. Another has been the fact that the majority of India’s population is not covered by any health insurance.

We describe the problems, including attrition faced by the Indian Institute of Head and Neck Oncology, in the central Indian state of Madhya Pradesh.

India is in the midst of a socio economic transformation. While improved health care has contributed to an increase in the average life span of its citizens, in the context of palliative care, the facilities and services available are inadequate. Less than 1% of India’s population has access to palliative care [1]. Anyone who has seen the agony that a patient can go through during the last stages of life and the consequent helplessness and trauma that the care givers experience, would immediately appreciate the importance of palliative care with its ‘high touch low tech approach’.

In 2010, the Medical Council of India accepted palliative care as a specialty [2]. However, the impact of that decision has still not percolated to a level that translates into easy access to palliative care for the common citizen.

Palliative care is in itself a rather difficult and demanding specialty and is known to take an emotional toll on medical personnel. Added to that is the fact that palliative medicine is not included in the undergraduate Medical curriculum [3] nor in the nursing curriculum. There is no systematic introduction to the specialty. Palliative care is still not a sought after specialty and when medical personnel do wish to train in palliative medicine they have to search for centres that provide this training.

The difficulties in offering palliative care in health care facilities in India have often been alluded to by doctors in meetings and conferences. The difficulties are numerous, from finding trained doctors, nursing staff and counselors, to the lack of readily available narcotic analgesic medications, their storage and appropriate dispensing. However, the availability of morphine has greatly improved in recent years [4].

According to the Directorate General of Health Services India, a national programme for palliative care as a centrally administered scheme is operational but 40% of the share of the grant must come from the states which have their own priorities and limitations. [5]

The Indian Institute of Head and Neck Oncology is a not-for-profit, charitable cancer facility situated in a village 15 km from the central Indian city of Indore. The centre is unique in that it has been developed to address the issue of India’s commonest cancers in men: head and neck cancers. Developed on the ten acres of land provided by the State Government at a nominal lease, the cost of construction has been supported by donors and the equipment gifted/received as grant-in-aid. These include the Indian teletherapy cobalt machine, Bhabhatron-II, and the indigenously developed Linear Accelerator, Siddharth-III, received as grant-in-aid from the Government, an acknowledgement of our charitable objectives.

Staffing and running expenses are not supported through such grants and ensuring the day-to-day running of the institute since 1995 has been a herculean task. Currently, we are basically a radiotherapy facility, treating on an average 35 patients per day, and we have successfully managed the radiotherapy treatment of more than 6079 patients, of which 1368 were given completely free treatment while partial charity was offered to 1640 patients.

The central Indian state of Madhya Pradesh, with a population of 72 598 thousand spread over 3,08,000 sq.km., has seven palliative care centres [6] including our own, a dauntingly small number. While we have been working in the field of palliative care since 1994, our hospice-cum-palliative care training facility was founded in 2013 [7]. The facility has 24 beds in the male and the female wings. It is equipped with the mandatory nursing stations, doctors’ offices, prayer/meditation room, and a kitchen facility. Besides this there is also an open to sky central courtyard, a space for patients and relatives to move around freely and enjoy the fresh air if they so wish. Although it is a separate building, the hospice is on the same campus as our cancer treatment centre, and patients are referred for palliative care without them having to travel any distance.

Before we actually commissioned our not-for-profit, charitable hospice and palliative care training centre, we had actively worked at sensitizing ourselves, our doctors and nurses as well as the medical community in general, hoping that this sensitized group would form the core of our institutional palliative care facility as well as spreading the message that terminally ill patients do not have to suffer and that there is a facility that can help improve quality of life in the last stage.

We were fortunate in that right from 1994 we got strong support from stalwarts in the field such as Dr Robert Twycross from the Oxford International Centre for Palliative Care and Ms Gilly Burn, a well-known palliative care nurse from the UK. These experts came to Indore in order to conduct palliative care awareness and training programmes and they also helped finance the training of our doctors in the UK and Kerala. This training enabled our doctors to learn and enhance their knowledge, equipping them to take up the challenge of a palliative care job and the responsibilities associated with it. In Indore, our well-structured programmes with bed-side training were open to medical personnel from the entire central Indian region.

Project based support also came in from time to time. The Oxford International Centre for Palliative Care (OICPC), the Australian Agency for International Development (AusAID), and the Indo American Cancer Association (IACA) all helped fund specific projects. On our part, proper and diligent utilization of funds thus received, helped spread our visibility, reputation and credibility. This has always ever remained our strength, something we have valued as a public charitable trust, as a group and as an Institution. Optimal utilization of financial grants received with stringent ‘control’ over expenses and reduced administrative costs.

Outreach activities at village level have been our forte, traveling that last mile to reach out to the most economically disadvantaged. We have conducted camps, in scores of government district hospitals and village level health care centres, to not only detect cancers but also to examine end stage patients, if any, and offer whatever relief we could.

AusAID helped us buy a mobile pain relief van and then in 1998/99 funded a pilot project on developing a district based palliative care model. It turned out to be a hugely successful learning experience for all concerned. Different levels of inputs were provided in training programmes to around 2000 primary health workers, nurses and doctors. The inputs were tailored to suit the work profile of the trainees. Primary health workers are the 1st level of the government district health delivery system. They are the ones in regular contact with people. So the key take away from the training, for primary health workers, was the importance of early detection of cancer and the right of the terminally ill cancer patient to receive palliative care and thus a better quality of life. The middle tier comprising nursing staff were given inputs keeping in mind the fact that it is they who would ensure that the instructions given by the doctor were being followed by the care givers of the terminally ill cancer patient.Then the doctors were given intensive 10-day training, at the end of which they were ready to offer palliative care to terminally ill cancer patients.

The response from the trainees was overwhelming. The doctors were immediately able to correlate the learning to their practice. A tremendous momentum was generated and all the trainees were imbued with a sense of purpose. All in all, a successful completion of the project. After seeing the achievements, Dr Robert Twycross was of the opinion that the model was replicable all over the country. The Dhar district administration of Madhya Pradesh was impressed with the quantum of work achieved. Unfortunately, funds were not available to ensure that the momentum achieved in Dhar district was sustained with systematic follow up along with refresher training, steps that would have ensured continued palliative care support at the doorsteps of patients living in the predominantly tribal district of Dhar that is incidentally adjacent to our institute.

## Practical difficulties in running a palliative care facility

What were the practical difficulties we faced once the palliative care centre was developed?

It is to be noted that we are involved in cancer care as a service without any consideration of profit. Despite the project based specific support to our palliative care programme, the pressure of woefully inadequate funds has always been a perennial challenge for us.

## Urban corporate migration: attrition

A trained palliative care physician is a precious and rare professional in India today. Naturally the issue of attrition is a huge challenge constantly facing facilities, especially for a financially constrained one like ours. With the growing number of large private hospitals coming up in the cities and towns needing their services, it is difficult to find a young physician, interested in and devoted to palliative medicine in its entirety and ready to work in a charitable organization with all its inherent handicaps. Private players in medical services are playing an increasing role in health care delivery in India. A physician trained in palliative care, by virtue of his/her sound background in pharmacodynamics, is an asset in any specialty that calls for symptom relief in the broad sense of the term and unless s/he is totally devoted to palliative care with service as a motive, his/her migration to other hospitals or even other medical specialties is a lurking possibility and uncertainty. The departure creates a huge void and leaves a negative impact. With ever increasing numbers of commercial hospitals offering lucrative financial packages with better growth prospects, young doctors are setting their sights on financially more rewarding prospects. The question also is, can palliative care offer the glamour and the lucre that cardiology, endocrinology or sports medicine does?

For us, problems arose after the physicians returned from palliative care training. In our set up, once a doctor is identified, s/he has to undergo training for the required enhancement/honing of clinical skills. We have sent our doctors all the way to UK and Kerala in the south of India, for several weeks of training in order to prepare them for the rigorous and emotionally demanding specialty that is palliative medicine. We tried to ensure that we chose the truly committed by selecting those that demonstrated keen interest and then putting them through several one-to-one counseling sessions. But, back from training, these physicians immediately became much sought after professionals for private hospitals, urban corporate set ups, much better funded hospitals offering better pay packages. Three doctors trained by us left, one went into private medical practice and refused to continue with palliative care on the ‘plea’ that he would then get a reputation of a doctor whose patients die; another left for the Middle East, and the third switched over to endocrinology after his demand for more salary was turned down.

This put us under continuous and tremendous pressure as there is a time lag of 12 to 18 months from the time a doctor is identified to the time the doctor can start contributing as an effective palliative care physician—searching for a suitable candidate, then sending the candidate for specialised training in palliative care, then came the settling in process on return.

As time went by, offering an appropriate solution did not assure a lasting solution. Perhaps, we need to get them to sign a bond? But both working for the economically disadvantaged as also working to improve the quality of life of a terminal stage patient is a calling and that cannot be enforced.

## Financial constraint

The other challenge is financial support that remains a critical issue for smooth running of any charitable centre, palliative care included. In the evolution of our project, we received support from: AusAID (Australian Agency for International Development) for our mobile palliative care van and for a pilot project on developing a district based model for palliative care; from OICPC (the Oxford International Centre for Palliative Care) for structured training programmes and staff salary; and from IACA (the Indo American Cancer Association) for meeting staff salaries. All were project specific, short duration. There is no denying that this support went a long way in helping us to spread awareness about palliative care and offer palliative care services. However, sustained effort in these areas was difficult if not impossible given the short term nature of such support coupled with our commitment of providing services to all, irrespective of their ability to pay.

The enormous challenge thus is to meet the day-to-day expenses with scant resources in view of our institutional philosophy of keeping our doors open to the needy.

Since it’s not easy to run a charitable palliative care centre without sustained financial support, ‘cross subsidization’ was a critical solution, with the affording patients beginning to pay some of their treatment costs. However, the cost of running the centre escalates every year and there is the maintenance to be thought of as well. Another issue is that quite often by the time a patient comes for palliative care, he or she has depleted their finances to the extent that charging even the nominal fees that we have in place makes it an ethical dilemma for us.

## Conclusion

Equipping, staffing and running such a centre is comparable to being in a ‘boxing ring’ all the time as there are crucial challenges in developing, running and expanding a charitable palliative care facility: lack of awareness about palliative care not only among patients and their care givers but also the medical fraternity, dearth of trained manpower with the consequent frequent job hopping by the trained manpower, paucity of funds.

However, we believe that palliative care is medicine at its best with its complete focus on sensitivity to the patient’s perspective. It goes beyond specialties and challenges the physician to strive for more. Obviously, the challenge is accentuated when one wants to ensure that all have access to palliative care, even in the far flung, remote areas. But the Indian Institute of Head and Neck Oncology, Indore Cancer Foundation Charitable Trust, is convinced that this is a cause worth supporting irrespective of the difficulties and it is committed to continue to face this challenge and sustained support of qualified medical personnel in a proper palliative care facility is an option that we will strive to pursue. As our AusAID supported project in Dhar district showed, if physicians in the peripheral health care system are better trained in the basics of palliative care then the need for institutional admissions will reduce with the added advantage of the terminally ill patient being able to spend the last days at home surrounded by family. In the Indian scenario, our strong familial ties and support makes this an eminently viable solution. The way forward continues to be training—training of more and more medical personnel as well as the patient’s care givers. A dependable network of mobile palliative care services, with home visits at required intervals is an option in countries like ours with areas of low development index settings.

Obviously, increased and sustained financial support will also be needed for the upkeep of the hospice and palliative care training facility and we will continue to strive for that as well.

Therefore, the possible solutions to the problem of attrition in palliative care are:

As attrition is an issue because of the huge shortage of trained personnel, the obvious answer is to increase trained personnel. The actual implementation is, however, not easy as this would involve making palliative care an integral part of medical curricula both for doctors and nurses that is decided at the level of medical or nursing council. So, this is clearly a long-term solution.

An interim solution is to offer more certificate courses in palliative care to doctors, nurses and para-medical staff especially for those in the mofussil areas.

Increasing mobile palliative care services for home visits would be another solution as the existing trained personnel would be in a position to reach out to a larger patient base.

Another solution lies in the closely-knit family structure in countries like India where the immediate family members are the first relay station. They can be empowered with training in home care of terminally ill patients. It will be emotionally acceptable and financially cost effective in the long run, after all preventing a bedsore would not only be better for the patient but would also be cheaper than treating it once it is developed.

Yet another solution would be to train and create a cadre of home care attendants. These attendants would not require high educational qualifications and so this solution has the added advantage of providing employment to the economically disadvantaged.

One more reason for shortage of manpower is the reality that palliative care is not a specialty that attracts talent. The obvious solution is if it becomes a financially rewarding specialty. Another way would be to highlight the benefits of knowing about palliative care across specialties, specifically symptom control and empathic communication. This would make learning palliative care attractive across specialties for doctors as well nurses.
